# Chemotherapy During Active SARS-CoV2 Infection: A Case Report and Review of the Literature

**DOI:** 10.3389/fonc.2021.662211

**Published:** 2021-04-12

**Authors:** Krzysztof Woźniak, Wojciech Sachs, Piotr Boguradzki, Grzegorz Władysław Basak, Rafał Stec

**Affiliations:** ^1^ Department of Oncology, Medical University of Warsaw, Warsaw, Poland; ^2^ Department of Hematology, Transplantation and Internal Medicine, Medical University of Warsaw, Warsaw, Poland

**Keywords:** COVID-19, PMBCL, colorectal cancer, mortality rate, chemotherapy, SARS-CoV-2

## Abstract

COVID-19 has become the biggest public health problem and one of the most important causes of death in many countries in the world. SARS-CoV-2 infection is most likely to be fatal in elderly patients with concomitant diseases. In this article we present two cases of asymptomatic SARS-CoV-2-positive patients suffering from cancer who were treated with chemotherapy. The first case, a patient with primary mediastinal B-cell lymphoma, shows that confirmed SARS-CoV-2 infection does not have to be a contraindication to chemotherapy. We describe the course of disease and discuss doubts related to the choice of chemotherapy regimen. The second patient was a male with metastatic sigmoid cancer treated with FOLFOX4 as first-line palliative chemotherapy. This case draws attention to asymptomatic SARS-CoV-2 carriers who underwent chemotherapy. Our patient was safely treated with chemotherapy without long break caused by viral infection. It should be remembered that there are asymptomatic carriers among cancer patients and that they may spread infection to others. On the other hand, delaying chemotherapy can cause rapid disease progression and reduce overall survival of our patients.

## Introduction

Coronavirus disease 2019 (COVID-19) is a disease known since December 2019, caused by SARS-CoV-2 virus. This acute respiratory disease was first noted in Wuhan, China and rapidly spread all over the world. On 11 March 2020, the World Health Organization (WHO) announced pandemic of SARS-CoV-2 infection. Since then COVID-19 has become the most prominent public health problem and one of the most important causes of death in many countries in the world. SARS-CoV-2 infection is most likely to be fatal in elderly patients with concomitant diseases (1). It has been proven that patients with cancer are more vulnerable to SARS-CoV-2 infection (2). They seem to be especially at risk of severe complications (3) and death due to COVID-19 (4–9). The worst prognosis concerns patients infected with COVID-19 and hematological malignancy (10–18). That is why treatment of cancer patient has become more difficult in pandemic era. Patients have to be protected from SARS-CoV-2 infection and at the same time they need treatment for cancer. It is known that most of SARS-CoV-2-positive patients have mild symptoms or no symptoms of the infection (19, 20). The crucial issue is how to deal with SARS-CoV-2-positive but asymptomatic oncological patients who require chemotherapy. There are limited data about the risks of chemotherapy and the effect of cytotoxic therapy on the course of viral infection. In this article we describe cases of two patients who underwent chemotherapy during SARS-CoV-2 infection and a review of available literature.

## Case 1

**Figure 1:**
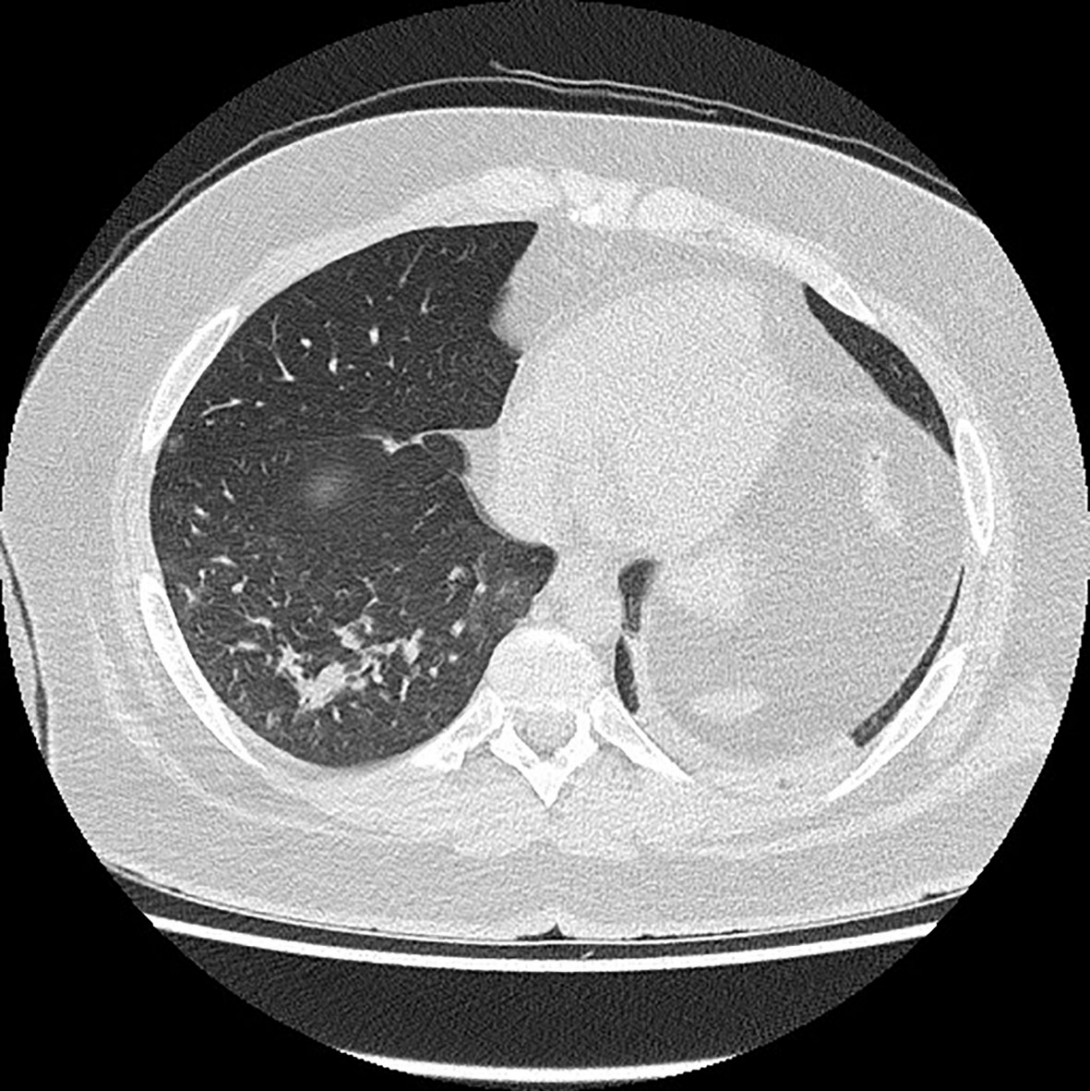
Chest computed tomography scan.

A 22-year-old patient without chronic diseases was admitted to the Department of Thoracic Surgery to establish diagnosis of mediastinal tumor. The first symptom of the disease was pathological fracture of the right arm. On admission, the patient’s performance status was good (ECOG score 1); he had no symptoms of infection. He complained of mild chest discomfort and hyperhidrosis, without fever. He denied dyspnea, cough, weight loss, loss of smell and taste. Blood oxygenation was normal (97%). Laboratory tests showed no abnormalities in peripheral blood count (WBC 7.27x10^9^/L, HGB 10.7 g/L, PLT 273 10^9^/L), but there was elevated level of LDH (1650 U/L). SARS-CoV-2 molecular nasal swab test before thoracorurgical procedure was negative. He had a biopsy of the mediastinal tumor, which confirmed primary mediastinal B-cell lymphoma (PMBCL): large B-lymphocytes, CD20+, CD23+/-, CD 30+/-, BCL6+, c-myc+, BCL2+ (double expressor), cyclD1-, Ki67 80%. Computed tomography scan revealed mediastinal tumor infiltrating thoracic wall, ascending aorta, pulmonary trunk. There were found two satellite tumors, 17 mm in diameter, in segments 4, 5 and 6 of the right lung; lymphoma tumors in the pancreas, pelvis, 10th right rib and proximal end of right humerus with pathological fracture. There was no lymphadenopathy detected. Active lymphoproliferative process was confirmed also in positron emission tomography scan (PET CT). The diagnosis was made of PMBCL stage IV according to Ann Arbour classification, aaIPI (age-adjusted international prognostic index) high intermediate (3 points). It was decided to perform orthopedic surgery of the pathological fracture and then to start chemotherapy. The SARS-CoV-2 molecular test was carried out before orthopedic treatment and it was positive. Date of positive SARS-CoV-2 test (twelve days after admission to hospital) suggests that hospital transmission of SARS-CoV-2 infection was the most probable. The patient was still in good performance status, without acute symptoms of COVID-19. There was no cough or fever detected. In the next CT scan (**Figure 1**), ground glass opacities in about 15% of the lungs were described – a typical imaging feature of SARS-CoV-2 infection. The patient was complaining of mild constant chest discomfort, but this symptom was present before SARS-CoV-2 infection and most probably caused by the presence of tumor mass in the mediastinum. Because of advanced stage PMBCL, it was decided to start lymphoma’s pretreatment. The patient was treated with 5-day regimen of cyclophosphamide 200 mg/m^2^/day and prednisone 40 mg/m^2^/day. We did not observe acute tumor lysis syndrome after this therapy. Laboratory tests showed normal level of LDH. Patient tolerated pretreatment very well without any side effects. No additional symptoms of SARS-CoV-2 infection appeared. Another SARS-CoV-2 test was also positive. It was decided to treat patient with chemotherapy according to CHOP-14 regimen (cyclophosphamide 750mg/m^2^, doxorubicin 50mg/m^2^, vincristin 1,4mg/m^2^, prednisone 40mg/m2). It was decided against the use of rituximab due to the risk of negative effect on SARS-CoV-2 infection. Chemotherapy was supported by G-CSF (filgrastim) administration and prophylaxis of tumor lysis syndrome. After two cycles of chemotherapy partial response of the lymphoma was observed in a CT scan. The patient complained of increased chest discomfort and tachycardia was detected. There were no abnormalities in ECG. Echocardiography examination showed myocardial injury with generalized hypokinesis and decreased ejection fraction (EF) of 45%, aortic valve regurgitation and tachycardia. Biochemical markers of myocardial injury – troponin I, NTproBNP and CK-MB mass – were within normal values. Cardiac drugs were administered (ACE inhibitor, beta-blocker) with good treatment results. The third and fourth cycles of chemotherapy were administered, with liposomal doxorubicin (Myocet) replacing doxorubicin in CHOP-14 regimen. The next SARS-CoV-2 test was negative. The patient is still continuing treatment with chemotherapy, since 4. cycle with rituximab (R-COMP).

## Case 2

The second patient is a 56-year-old man with sigmoid cancer and unresectable liver metastases treated with the first line of palliative chemotherapy with FOLFOX4 regimen. The patient was in good performing status (ECOG 1), complaining of polyneuropathy grade 1 after oxaliplatin treatment. He had coronary heart disease; he underwent myocardial infarction in 2015, with coronary artery angioplasty (PTCA). He underwent sigmoidectomy in November 2019 and was diagnosed with sigmoid adenocarcinoma (pT3N1b). Examination of tumor tissue detected KRAS mutation in codon 12. In January 2020 he started chemotherapy with FOLFOX4 regimen. Due to polyneuropathy the dose of oxaliplatin has been reduced to 65mg/m^2^.

Between 10.11.2020 and 12.11.2020 the patient administrated 21st. cycle of FOLFOX4. From 14.11.2020 to 27.11.2020 the patient was in quarantine because his daughter was tested positive of SARS-CoV-2 infection.

He had no symptoms of infection. On 24.11.2020 he underwent genetic SARS-CoV-2 test, which gave negative result. On 06.12.202 SARS-CoV-2 antibodies level was measured. This investigation revealed positive antibody levels: IgM 4.54 s/c (>0.99 considered positive) and IgG 4.16 (>1.40 considered positive). This shows that our patient had asymptomatic SARS-CoV-2 infection during chemotherapy. The most probable source of infection was patient’s daughter. It is also highly possible that patient underwent SARS-CoV-2 infection in the time of quarantine, but it is not possible to prove the way of transmission and the exact time of infection. In this situation hospital transmission of SARS-CoV-2 infection is less probable, but cannot be excluded. The next cycle of chemotherapy had to be postponed to a week later because of 3rd grade neutropenia. The patient is still continuing chemotherapy without any new side effects and complications.

## Discussion

Primary mediastinal B-cell lymphoma (PMBCL) is a rare type of aggressive lymphoma. It constitutes about 3% of non-Hodgkin lymphomas and 6–10% of diffuse large B-cell lymphomas (21) Prognosis is relatively good – 5-year overall survival (OS) is 93–98% in early stage of disease and 70–75% in the group of patients with negative prognostic factors (21). According to ESMO Guidelines (22) the R-CHOP chemotherapy regimen remains a standard therapy. More intensive regimens such as DA-EPOCH-R could be considered depending on a center’s experience. In many centers also other regimens such as dose-dense R-CHOP, R-V/MACOP-B or GMALL are used.

The role of immunosuppression during SARS-CoV-2 infection is unclear. It has been proven that small doses of dexamethasone reduce mortality in severe COVID-19 by stopping autoimmune destruction of the lungs (so called “cytokine storm”) (23). There were some observations suggesting that steroids shorten period of oxygen dependence and improve disease course (24). It has been proven in an open randomized trial that dexamethasone (at a dose of 6mg daily) reduces 28-day mortality in patients hospitalized due to SARS-CoV-2 (25). These results form the basis of many clinical guidelines, also Polish ones, which recommend dexamethasone in the treatment of patients with COVID-19 (26). However, we must remember that there are a lot of data suggesting that immunosuppression in early stage of COVID-19 is not recommended.

Another drug, which is considered as a useful treatment to prevent aggressive autoimmune reaction, is cyclophosphamide. Cyclophosphamide is a part of many chemotherapy regimens such as CHOP, which was administered to our patient. Cyclophosphamide is also used as immunosuppressive drug after haploidentical stem-cell transplantation or other organ transplantations and is widely used in rheumatological disorders. There are attempts to use cyclophosphamide in cytokine storm prevention (27). A case of a young man who underwent COVID-19 simultaneously with immunosuppressive regimen containing cyclophosphamide has been reported. The authors suspect that cyclophosphamide could prevent this patient from developing severe course of pulmonary infection (28). Nowadays cyclophosphamide is not used as a standard SARS-CoV-2 therapy.

There are limited data suggesting that other immunosuppressive drugs, e.g. rituximab, can worsen the course of SARS-CoV-2 infection. Rituximab is a monoclonal anti-CD20 antibody that causes depletion of peripheral B lymphocytes. This leads to severe humoral and cellular immunodeficiency. Some cases of persistent SARS-CoV-2 viremia in patients after rituximab treatment have been reported (29). Yasuda et al. described a case of patient with focal lymphoma after recent rituximab treatment that did not develop SARS-CoV-2 antibodies and died due to persistent pneumonia (30). Rituximab can also prolong incubation period of SARS-CoV-2 infection (>21 days), which could facilitate spreading of infection in a population (31). On the other hand there are data showing that the mortality rate of patients with COVID-19 treated with rituximab maintenance therapy after remission in follicular lymphoma is lower than when combined with chemotherapy (10): 31% vs 47%. However, it is still much higher than in general population. Because of the risk of serious course of SARS-CoV-2 infection our patient did not receive chemotherapy with rituximab.

Our patient developed cardiac insufficiency during PMBCL chemotherapy. The reason of decreased ejection fraction (EF) is unclear. Its pathogenesis seems to be multicausal. Chemotherapy with anthracycline can be a reason of heart failure even if the dose is lower that maximum dose. SARS-CoV-2 infection itself is frequently related to cardiac manifestations. Cardiac involvement manifesting as troponin level elevation is observed in 20–30% of patients admitted to hospital due to COVID-19 (32, 33). SARS-CoV-2 infection causes pathological changes not only in the respiratory tract but also in other organs. It causes systemic inflammation, which plays role in endothelial damage also in the cardiovascular system. In this way, it leads to myocarditis, cardiac fibrosis and pericarditis (33), which are responsible for a poor prognosis in COVID-19 patients. In the case of our patient, it is impossible to prove which mechanism played a key role in heart failure, but it is unlikely that SARS-CoV-2 infection damaged myocardium without any lesions in the lungs.

Neither of our patients had leukocytosis or lymphopenia detected in blood count (which are typical for COVID-19) before and after SARS-CoV-2 infection. This is similar to other studies (34) in which all asymptomatic patients had normal level of lymphocytes. Probably it indicates that the asymptomatic carrier’s immunity is not significantly disturbed by COVID-19. In our second case, neutropenia appeared after infection and was not related to chemotherapy. Usually, neutropenia appears in severe stage of COVID-19 infection and is connected with higher mortality (35), but it has not been reported in asymptomatic SARS-CoV-2 carriers.

Another issue is asymptomatic lesions in lungs in CT scan, which were detected in our first patients. This situation is reported in about 30% of asymptomatic SARS-CoV-2 positive patients (36).

Asymptomatic carriers are a crucial problem in COVID-19 propagation. It is suspected that they have the same infective potential as people with symptoms (2). This fact is dangerous for cancer patients undergoing chemotherapy because this group is especially susceptible to severe course of COVID-19 (37).

On the other hand, as it has been shown in our cases, patients with asymptomatic SARS-CoV-2 infection can be safely treated with chemotherapy.

Similar observations were done by Hempel et al. (38). He showed that most SARS-CoV-2-infected patients were asymptomatic and the infection had no effect on chemotherapy (38). In contrast to this observation, one meta-analysis showed that chemotherapy during COVID-19 (or within 30 days before COVID-19) increased the risk of death due to COVID-19 (39). This paper did not prove that immunotherapy, targeted therapies, cancer surgery or radiotherapy increase the mortality rate or severe course of COVID-19.

There are data available from Kuderer’s study (6) showing that mortality rate due to COVID-19 in population of cancer patients is 13% and it is significantly higher than in general population. Only 4% of patients in this group had no symptoms of SARS-CoV-2 infection. It was proven that worse prognosis had elderly patients, with worse ECOG (Eastern Cooperative Oncology Group) performance status (>1), with progressive cancer disease.

Lee et al. (4) has shown that after adjusting for known risk factors (age, gender, and comorbidities), chemotherapy in the past 4 weeks had no significant effect on mortality from COVID-19 disease, in comparison to patients without recent chemotherapy.

There are also data published which suggest that worse prognosis of patients with solid tumors is connected with known risk factors only, such as age and comorbidities. In Ruthrich’s paper (7) mortality rate of cancer patients was higher than in non-cancer group (22,5% vs 14%) but after adjustments for other risk factors, mortality was comparable.

There is growing number of papers which proves that prognosis of COVID-19 is very poor in group of patients with hematologic cancer (Tab. 1). Vijenthira et al. in metaanalysis (10) has shown that risk of death due to COVID-19 in group of hospitalized hematologic malignancies patients is 34% and it is much higher than in general population. In this group the highest risk of death had patients with acquired bone marrow failure syndromes (53%), acute leukemias (41%), plasma cell dyscrasias (33%) and lymphomas (32%).

Similarly, very high mortality rate (37%) was shown by Passamonti et al. (11). In this paper the following independent risk factors of death are identified: elderly age, progressive disease, neoplasm type- myeloid leukemia, aggressive non-Hodgking lymphoma, indolent lymphoma.

In the analysis of 264 patients published by the ASH Research Collaborative Data Hub (12), mortality rate was comparable to the other studies (30%). Authors pay attention to the group of patients with pre-COVID prognosis of <12 months (due to the hematologic malignancy), who have especially unfavorable course of COVID-19. These patients had higher risk of moderate or severe COVID-19 and more often were not admitted to intensive care unit in favor of palliative treatment. It contributes to the very poor outcome in this group.

In some retrospective studies, the mortality rate in the hematologic cancer patients cohort was as high as 50%, and it was even more than twice higher than with the case of solid tumors (13). Such poor outcomes can be seen in cohort of hospitalized patients only, mostly with poor prognosis before SARS-Co-V2 infection. Slightly lower death rate reports Yang (14). In his paper it was shown that chemotherapy in the last four weeks before COVID-19 was an independent death risk factor.

Yigenoglu (15) has shown fatality rate of 13.8% compared to 6.8% in the group of patients without cancer. These results are much lower than in similar studies concerning patients with hematologic malignancies. However, the mortality rate proportion between the cancer patients and the non-cancer patients was comparable to the other studies. Oncological patients statistically more often underwent severe COVID-19 and were hospitalized in ICU. The explanation of these results can be a fact that the study group, in contrast to any other analyses, constituted mostly of the non-hospitalized patients.

Our two case reports show that chemotherapy can be administered during SARS-CoV-2 infection. Decision about systemic treatment in SARS-CoV-2-positive patients should be made by multidisciplinary team after consideration of all treatment options and risks of chemotherapy. The development of symptomatic SARS-CoV-2 infection (COVID-19 disease) during chemotherapy has to be taken into account in pandemic era.

## Author Contributions

KW contributed to design of the study and wrote the first draft of the manuscript. RS contributed to conception of the study and contributed to the revision. PB contributed to design discussion and revision of the manuscript. WS was collecting the clinical data and contributed to critical revision of the manuscript. GB contributed to the manuscript revision. All authors contributed to the article and approved the submitted version.

## Conflict of Interest

The authors declare that the research was conducted in the absence of any commercial or financial relationships that could be constructed as a potential conflict of interest.

## References

[1] Guan WJ Ni ZY Hu Y Liang WH Ou CQ He JX. Clinical Characteristics of Coronavirus Disease 2019 in China. N Engl J Med (2020) 382(18):1708–20. 10.1056/NEJMoa2002032 PMC7092819 32109013

[2] Dai M Liu D Liu M Zhou F Li G Chen Z. Patients with Cancer Appear More Vulnerable to SARS-CoV-2: A Multicenter Study during the COVID-19 Outbreak. Cancer Discovery (2020) 10(6):783–91. 10.1158/2159-8290.CD-20-0422 PMC7309152 32345594

[3] Liang W Guan W Chen R Wang W Li J Xu K. Cancer patients in SARS-CoV-2 infection: a nationwide analysis in China. Lancet Oncol (2020) 21(3):335–7. 10.1016/S1470-2045(20)30096-6 PMC7159000 32066541

[4] Lee LY Cazier JB Angelis V Arnold R Bisht V Campton NA. COVID-19 mortality in patients with cancer on chemotherapy or other anticancer treatments: a prospective cohort study published correction appears in Lancet. 2020 Aug 22;396(10250):534. Lancet (2020) 395(10241):1919–26. 10.1016/S0140-6736(20)31173-9 PMC7255715 32473682

[5] Garassino MC Whisenant JG Huang LC Trama A Torri V Agustoni F. COVID-19 in patients with thoracic malignancies (TERAVOLT): first results of an international, registry-based, cohort study. Lancet Oncol (2020) 21(7):914–22. 10.1016/S1470-2045(20)30314-4 PMC7292610 32539942

[6] Kuderer NM Choueiri TK Shah DP Shyr Y Rubinstein SM Rivera DR. Clinical impact of COVID-19 on patients with cancer (CCC19): a cohort study published correction appears in Lancet. 2020 Sep 12;396(10253):758. Lancet (2020) 395(10241):1907–18. 10.1016/S0140-6736(20)31187-9 PMC7255743 32473681

[7] Rüthrich MM Giessen-Jung C Borgmann S Classen AY Dolff S Gruner B. COVID-19 in cancer patients: clinical characteristics and outcome-an analysis of the LEOSS registry. Ann Hematol (2021) 100(2):383–93. 10.1007/s00277-020-04328-4 PMC7648543 33159569

[8] Miyashita H Mikami T Chopra N Yamada T Chernyavsky S Rizk D. Do patients with cancer have a poorer prognosis of COVID-19? An experience in New York City. Ann Oncol (2020) 31(8):1088–9. 10.1016/j.annonc.2020.04.006 PMC7172785 32330541

[9] Mehta V Goel S Kabarriti R Cole D Goldfinger M Acuna-Villaorduna A. Case Fatality Rate of Cancer Patients with COVID-19 in a New York Hospital System. Cancer Discovery (2020) 10(7):935–41. 10.1158/2159-8290.CD-20-0516 PMC7334098 32357994

[10] Vijenthira A Gong IY Fox TA Booth S Cook G Fattizzo B. Outcomes of patients with hematologic malignancies and COVID-19: a systematic review and meta-analysis of 3377 patients. Blood (2020) 136(25):2881–92. 10.1182/blood.2020008824 PMC7746126 33113551

[11] Passamonti F Cattaneo C Arcaini L Bruna R Cavo M Merli F. Clinical characteristics and risk factors associated with COVID-19 severity in patients with haematological malignancies in Italy: a retrospective, multicentre, cohort study. Lancet Haematol (2020) 7(10):e737–45. 10.1016/S2352-3026(20)30251-9 PMC7426107 32798473

[12] Wood WA Neuberg DS Thompson JC Tallman MS Sekeres A Sehn LH. Outcomes of patients with hematologic malignancies and COVID-19: A report from the ASH Research Collaborative Data Hub. Blood Adv (2020) 4:5966–75. 10.1182/bloodadvances.2020003170 PMC7724912 33278301

[13] Meng Y Lu W Guo E Liu J Yang B Wu P. Cancer history is an independent risk factor for mortality in hospitalized COVID-19 patients: a propensity score-matched analysis. J Hematol Oncol (2020) 13(1):75. 10.1186/s13045-020-00907-0 32522278 PMC7286218

[14] Yang K Sheng Y Huang C Jin Y Xiong N Jiang K. Clinical characteristics, outcomes, and risk factors for mortality in patients with cancer and COVID-19 in Hubei, China: a multicentre, retrospective, cohort study. Lancet Oncol (2020) 21(7):904–13. 10.1016/S1470-2045(20)30310-7 PMC7259917 32479787

[15] Yigenoglu TN Ata N Altuntas F Basci S Dal MS Korkmaz S. The outcome of COVID-19 in patients with hematological malignancy. J Med Virol (2021) 93(2):1099–104. 10.1002/jmv.26404 PMC7436524 32776581

[16] Hultcrantz M Richter J Rosenbaum C Patel D Smith E Korde N. COVID-19 infections and outcomes in patients with multiple myeloma in New York City: a cohort study from five academic centers. Preprint MedRxiv (2020) 2020.06.09.20126516. 10.1101/2020.06.09.20126516 PMC8510790 34661147

[17] Mato AR Roeker LE Lamanna N Allan JN Leslie L Pagel JM. Outcomes of COVID-19 in patients with CLL: a multicenter international experience. Blood (2020) 136(10):1134–43. 10.1182/blood.2020006965 PMC7472711 32688395

[18] Scarfò L Chatzikonstantinou T Rigolin GM Quaresmini G Motta M Vitale C. COVID-19 severity and mortality in patients with chronic lymphocytic leukemia: a joint study by ERIC, the European Research Initiative on CLL, and CLL Campus. Leukemia (2020) 34(9):2354–63. 10.1038/s41375-020-0959-x PMC7347048 32647324

[19] Oran DP Topol EJ. Prevalence of Asymptomatic SARS-CoV-2 Infection : A Narrative Review. Ann Intern Med (2020) 173(5):362–7. 10.7326/M20-3012 PMC7281624 32491919

[20] Nikolai LA Meyer CG Kremsner PG Velavan TP. Asymptomatic SARS Coronavirus 2 infection: Invisible yet invincible. Int J Infect Dis (2020) 100:112–6. 10.1016/j.ijid.2020.08.076 PMC7470698 32891737

[21] Dabrowska-Iwanicka A Walewski JA. Primary mediastinal large B-cell lymphoma. Curr Hematol Malig Rep (2014) 9(3):273–83. 10.1007/s11899-014-0219-0 PMC4180024 24952250

[22] Vitolo U Seymour JF Martelli M Illerhaus G Illidge T Zucca E. Extranodal diffuse large B-cell lymphoma (DLBCL) and primary mediastinal B-cell lymphoma: ESMO Clinical Practice Guidelines for diagnosis, treatment and follow-up. Ann Oncol (2016) 27(suppl 5):v91–v102. 10.1093/annonc/mdw175 27377716

[23] Theoharides TC Conti P. Dexamethasone for COVID-19? Not so fast. J Biol Regul Homeost Agents (2020) 34(3):1241–3. 10.23812/20-EDITORIAL_1-5 32551464

[24] Wang Y Jiang W He Q Wang C Wang B Zhou P. A retrospective cohort study of methylprednisolone therapy in severe patients with COVID-19 pneumonia. Signal Transd Targ Ther (2020) 5(1):57. 10.1038/s41392-020-0158-2 PMC7186116 32341331

[25] RECOVERY Collaborative Group Horby P Lim WS Emberson JR Mafham M Bell JL. Dexamethasone in Hospitalized Patients with Covid-19 - PreliminaryReport published online ahead of print, 2020 Jul 17. N Engl J Med (2020) 384(8):693–704. 10.1056/NEJMoa2021436 32678530 PMC7383595

[26] Flisiak R Parczewski M Horban A Jaroszewicz J Kozielewicz D Pawłowska M. Management of SARS-CoV-2 infection: recommendations of the Polish Association of Epidemiologists and Infectiologists. Annex no. 2 as of October 13, 2020. Pol Arch Intern Med (2020) 130(10):915–8. 10.20452/pamw.15658 33119223

[27] Revannasiddaiah S Kumar Devadas S Palassery R Kumar Pant N Maka VV. A potential role for cyclophosphamide in the mitigation of acute respiratory distress syndrome among patients with SARS-CoV-2. Med Hypotheses (2020) 144:109850. 10.1016/j.mehy.2020.109850 32526511 PMC7244432

[28] Moeinzadeh F Dezfouli M Naimi A Shahidi S Moradi H. Newly Diagnosed Glomerulonephritis During COVID-19 Infection Undergoing Immunosuppression Therapy, a Case Report. Iran J Kidney Dis (2020) 14(3):239–42. 32361703

[29] Tepasse PR Hafezi W Lutz M Kuhn J Wilms C Wiewrodt R. Persisting SARS-CoV-2 viraemia after rituximab therapy: two cases with fatal outcome and a review of the literature. Br J Haematol (2020) 190(2):185–8. 10.1111/bjh.16896 PMC7300950 32557623

[30] Yasuda H Tsukune Y Watanabe N Sugimoto K Uchimura A Tateyama M. Persistent COVID-19 Pneumonia and Failure to Develop Anti-SARS-CoV-2 Antibodies During Rituximab Maintenance Therapy for Follicular Lymphoma. Clin Lymphoma Myeloma Leuk (2020) 20(11):774–6. 10.1016/j.clml.2020.08.017 PMC7442568 32933879

[31] Koff AG Laurent-Rolle M Hsu JC Malinis M. Prolonged incubation of severe acute respiratory syndrome coronavirus 2 (SARS-CoV-2) in a patient on rituximab therapy published online ahead of print, 2020 Oct 7. Infect Control Hosp Epidemiol (2020) 1–2. 10.1017/ice.2020.1239 PMC7578652 33023685

[32] Shi S Qin M Shen B Cai Y Liu T Yang F. Association of Cardiac Injury With Mortality in Hospitalized Patients With COVID-19 in Wuhan, China. JAMA Cardiol (2020) 5(7):802–10. 10.1001/jamacardio.2020.0950 PMC7097841 32211816

[33] Knockaert DC. Cardiac involvement in systemic inflammatory diseases. Eur Heart J (2007) 28(15):1797–804. 10.1093/eurheartj/ehm193 17562669

[34] Huang Q Hu S Ran FM Liang T-J Wang H-X Chen C-C. Asymptomatic COVID-19 infection in patients with cancer at a cancer-specialized hospital in Wuhan, China - Preliminary results. Eur Rev Med Pharmacol Sci (2020) 24(18):9760–4. 10.26355/eurrev_202009_23070 33015823

[35] Yarza R Bover M Paredes D Lopez-Lopez F Jara-Casas D Castelo-Loureiro A. SARS-CoV-2 infection in cancer patients undergoing active treatment: analysis of clinical features and predictive factors for severe respiratory failure and death. Eur J Cancer (2020) 135:242–50. 10.1016/j.ejca.2020.06.001 PMC7275164 32586724

[36] Varble N Blain M Kassin M Xu S Turkbey EB Amalou A. CT and clinical assessment in asymptomatic and pre-symptomatic patients with early SARS-CoV-2 in outbreak settings published online ahead of print, 2020 Nov 4. published correction appears in Eur Radiol. 2020 Dec 8;. Eur Radiol (2020) 1–12. 10.1007/s00330-020-07401-8

[37] ElGohary GM Hashmi S Styczynski J Kharfan-Dabaja MA Alblooshi RM de la Cámara R. The risk and prognosis of COVID-19 infection in cancer patients: Asystematic review and meta-analysis published online ahead of print, 2020 Jul 30.Hematol Oncol Stem Cell Ther (2020)S1658–3876(20) 30122–9. 10.1016/j.hemonc.2020.07.005 PMC7390725 32745466

[38] Hempel L Piehler A Pfaffl MW Molnar J Kirchner B Robert S. SARS-CoV-2 infections in cancer outpatients-Most infected patients are asymptomatic carriers without impact on chemotherapy. Cancer Med (2020) 9(21):8020–8. 10.1002/cam4.3435 PMC7643635 33022856

[39] Yekedüz E Utkan G Ürün Y. A systematic review and meta-analysis: the effect of active cancer treatment on severity of COVID-19. Eur J Cancer (2020) 141:92–104. 10.1016/j.ejca.2020.09.028 33130550 PMC7538140

